# Concomitant deletion of chromosome 16p13.11 and triplication of chromosome 19p13.3 in a child with developmental disorders, intellectual disability, and epilepsy

**DOI:** 10.1186/s13039-015-0115-x

**Published:** 2015-02-05

**Authors:** Elisa Tassano, Lucia Rosaia De Santis, Maria Franca Corona, Stefano Parmigiani, Dalila Zanetti, Simona Porta, Giorgio Gimelli, Cristina Cuoco

**Affiliations:** Laboratorio di Citogenetica, Istituto G.Gaslini, L.go G.Gaslini 5, 16147 Genoa, Italy; SSD Genetica Medica, Ospedale S. Andrea, La Spezia, Italy; SS Neonatologia Fisiologica, Ospedale S. Andrea, La Spezia, Italy; SC Pediatria e Neonatologia, Ospedale S. Andrea, La Spezia, Italy

**Keywords:** Deletion, 16p13.11, Triplication, 19p13.3, Array-CGH, Developmental disorders, Intellectual disability, Epilepsy

## Abstract

**Background:**

Rare copy number variations (CNVs) are today recognized as an important cause of various neurodevelopmental disorders, including mental retardation and epilepsy. In some cases, a second CNV may contribute to a more severe clinical presentation.

**Results:**

Here we describe a patient with epilepsy, mental retardation, developmental disorders, and dysmorphic features, who inherited a deletion of 16p13.11 and a triplication of 19p13.3 from his father and mother, respectively. The mother presented mild mental retardation and language delay too.

**Conclusions:**

We discuss the phenotypic consequences of the two CNVs and suggest that their synergistic effect is likely responsible for the complicated clinical features observed in our patient.

## Background

Rare copy number variations (CNVs) are today recognized as important genetic causes or risk factors for intellectual disability (ID), autism, schizophrenia, and epilepsy.

Recurrent deletions and duplications are important contributors to neurodevelopmental disorders with complex inheritance, including epilepsy. Among them, 16p13.11 deletion was described in individuals with schizophrenia [1], autism [2], epilepsy [3-5], attention deficit hyperactivity disorders [6], intellectual disability, microcephaly, and/or multiple congenital anomalies [2,7]. The variety of phenotypes and the presence of the rearrangement in unaffected relatives may be due to several factors, such as incomplete penetrance, variable expressivity, and failure to recognize subtle manifestations, imprinting, and environmental factors.

In a number of patients, the phenotypic differences could be due to a second CNV, as reported in a study on 2312 children known to carry a CNV associated with intellectual disability and congenital abnormalities [8]. Furthermore, this study revealed that the secondary CNVs are preferentially transmitted from maternal carriers.

Interstitial duplications, and even more triplications, of the short arm of chromosome 19 are very rare. To our knowledge, only four patients have been described with a pure interstitial duplication of 19p ranging from the locus 19p13.2 to 19p13.3 and presenting various phenotypic features [9-12].

We describe here, for the first time, clinical observations and array-CGH analysis of a patient with concomitant occurrence of a deletion of chromosome 16p13.11 and a triplication of chromosome 19p13.3.

### Clinical report

The patient is a 13-year-old Italian male born to non-consanguineous parents with no other siblings. The mother presented with mild mental retardation and language delay. The father had a sternal malformation described as pectus excavatum and his language was poor. The infant was delivered at 37 weeks of gestation by caesarean section due to polyhydramnios after an uneventful pregnancy. At birth, his weight, length, and occipitofrontal circumference (OFC) were normal (>50th centile). He presented a dermoid cyst in his left eyebrow and bilateral clubfoot. Since the first years of life, he showed psychomotor development and language delay.

CT scan and brain MRI showed an arachnoid cyst on the right side in the middle cranial fossa associated with temporo-mesial hypoplasia. An expansion of the cyst reached the paraclinoid region. Until the age of 7 years he was fed with liquid food because of difficulties in swallowing and chewing solid foods. Control of the sphincters was achieved at 7 years of age.

**Figure 1:**
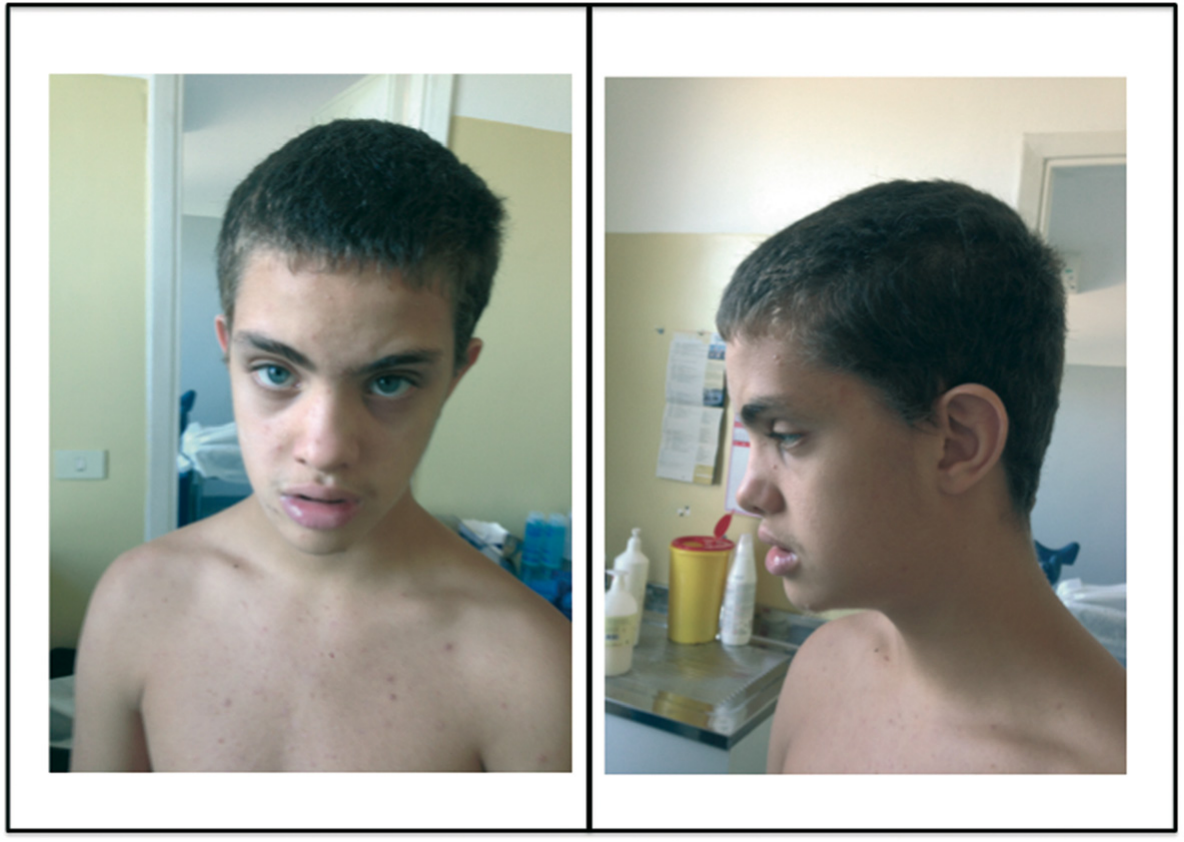
**Patient at the age of 13 years old.** Note long coarse face, flat occiput, brachycephaly, hypertelorism, shortened palpebral fissures, broad nasal bridge and short nose, long philtrum, full prominent everted lower lips, open mouth, low set ears, maxillary hypoplasia.

At 13 years of age, he was admitted to our hospital for seizure in apyrexia. His stature and OFC were normal. He had dysmorphic features like a long coarse face, flat occiput, brachycephaly, hypertelorism, shortened palpebral fissures, broad nasal bridge, short nose, long philtrum, prominent everted lower lips, open mouth, low set ears, maxillary hypoplasia, brachydactyly, and talipes equinovarus (Figure 1). Moreover, he had a developmental disorder characterized by poor speech and language delay. Neuropsychiatric evaluation showed intellectual disability with hyperactive and aggressive behaviour.

Eye examination and fundoscopy revealed a right optic papilla with normal macula and the presence of tissue oedema at the upper pole. EEG showed an abnormal generalized non-specific pattern, right epileptiform abnormal pattern waveform with spikes and spike and waves discharges.

## Results

**Figure 2:**
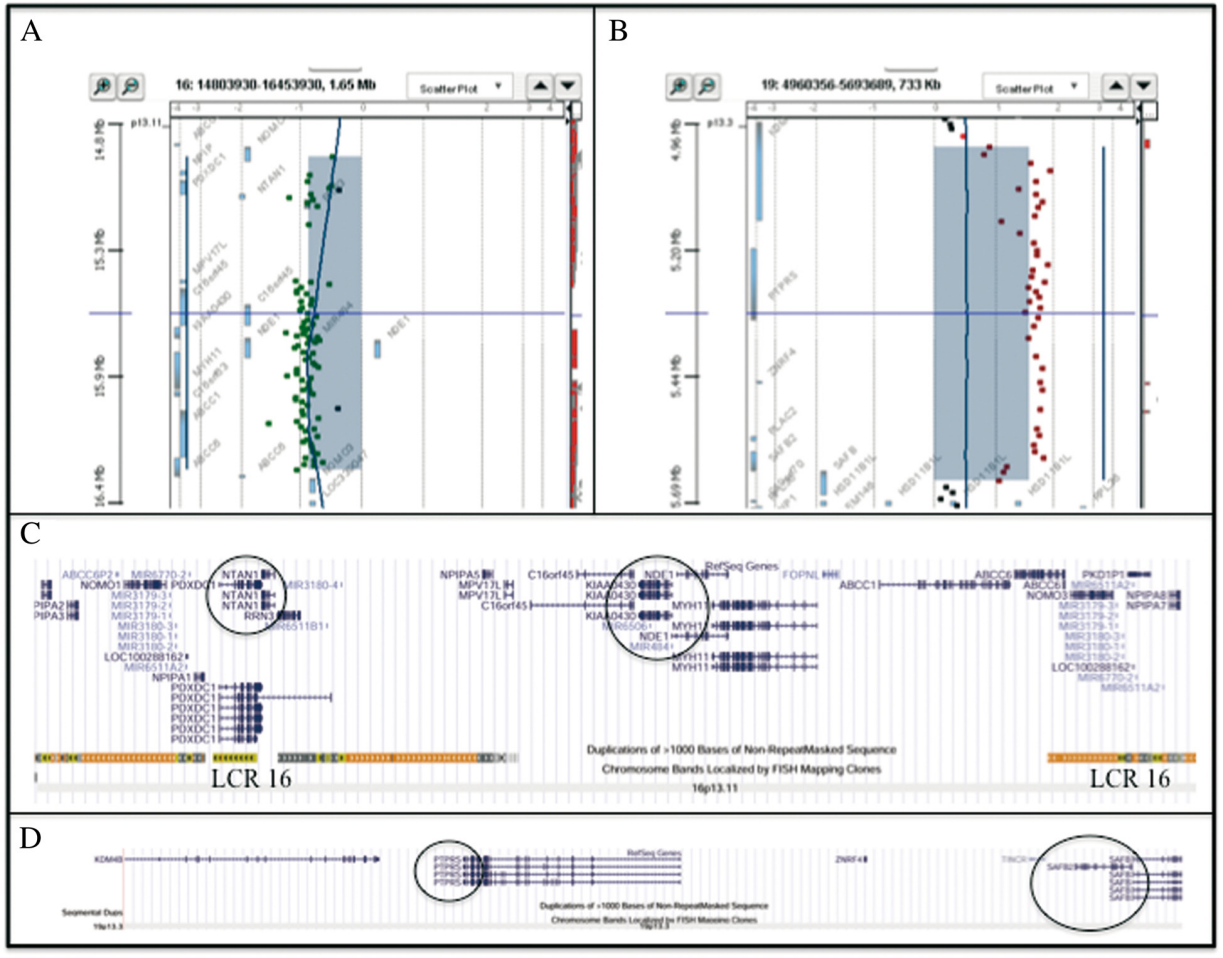
**Results of array-CGH analysis. A)** Array-CGH analysis shows a 1.323 Mb interstitial deletion at 16p13.3 band spanning from probe A_16_P40566850 (14,968,855 bps) to probe A_16_P03120838 (16,292,235 bps) inherited from the father. **B)** Array-CGH analysis shows a a triplication of 625.8Kb at 19p13.3 band spanning from probe A_16_P20946356 (5,016,071 bps) to probe A_14_P124343 (5,641,847 bps), inherited from his mother. **C)** Overview of the region 16p13.11 and its gene contents, according to the UCSC Genome Browser (GRCh37/hg19 assembly). The circles indicate the genes, which could be responsible for the phenotypic features of the 16p13.11 deletion patients. The deleted region was flanked by low copy repeats (LCR16s). **D)** Overview of the region 19p13.3 and its gene contents, according to the UCSC Genome Browser (GRCh37/hg19 assembly). The circles indicate the genes, which could be responsible for the phenotypic features of the patient with the 19p13.3 triplication.

Cytogenetic analysis, performed on GTG-banded metaphases from cultured lymphocytes of the patient and his parents, showed normal karyotypes. Considering the phenotypic abnormalities of the patient, array-CGH analysis was performed, revealing two CNVs. The first was a 1.323 Mb interstitial deletion at 16p13.11 spanning from probe A_16_P40566850 (14,968,855 bps) to probe A_16_P03120838 (16,292,235 bps), inherited from the father; the deleted region was flanked by low copy repeats (LCR16s). The second was a triplication of 625.8Kb at 19p13.3 spanning from probe A_16_P20946356 (5,016,071 bps) to probe A_14_P124343 (5,641,847 bps), inherited from the mother (Figure 2A-B) [arr 16p13.11(14,968,855-16,292,235)×1 pat, 19p13.3(5,016,071-5,641,847)×4 mat]. Array-CGH of maternal grandmother and maternal aunt were normal. FISH analysis confirmed the duplication on 19p13.3 of the proband and his mother excluding any insertion or translocation.

The 16p13.3 deleted region contains nine OMIM genes: *NOMO1* (MIM 609157) nodal modulator 1; *PDXDC1* (MIM 614244) pyridoxal-dependent decarboxylase domain-containing protein 1; *NTAN1* (MIM 615367) n-terminal asparagine amidase; *RRN3* (MIM 605121) *RRN3*, s. cerevisiae, homolog; *KIAA0430* (MIM 614593) KIAA0430 gene; *NDE1* (MIM 609449) Nude, homolog of A. nidulans; *MYH11* (MIM 160745) myosin, heavy chain 11, smooth muscle; *ABCC1* (MIM 158343) ATP-binding cassette, subfamily c, member1; *ABCC6* (MIM 603234) ATP-binding cassette, subfamily c, member 6. This region also contains twelve micro RNA (Figure 2C). The trisomic region of chromosome 19 harbours six OMIM genes: *KDM4B* (MIM 609765) lysine-specific demethylase 4B; *PTPRS* (MIM 601576) protein-tyrosine phosphatase, receptor-type, sigma; *ZNRF4* (MIM 612063) zinc finger and ring finger protein 4; *TINCR* (MIM 615241) terminal differentiation-induced noncoding RNA; *SAFB2* (MIM 608066) scaffold attachment factor b2; *SAFB1* (MIM 602895) scaffold attachment factor b (Figure 2D).

## Discussion

We describe a boy with a paternally inherited interstitial deletion at 16p13.11 band and a concomitant partial interstitial triplication of 19p13.3 inherited from his mother. Essentially, the boy presented cerebral, skeletal, neurological anomalies, and facial dysmorphisms. Furthermore, he had intellectual disability with severe speech and language delay associated with hyperactive and aggressive behaviour.

Although genomic imbalances involving 16p13.11 have already been reported in the literature, this is the first reported patient carrying a 16p13.11 microdeletion and a concomitant 19p13.3 triplication.

The recurrent 16p13.11 microdeletion spans about 1.65 Mb encompassing 15 RefSeq genes. Breakpoints of the deletion are located in two LCR blocks (LCR16s), which implies that non-allelic homologous recombination (NAHR) between these flanking LCRs may mediate this rearrangements.

Among deleted genes in the 16p13.11 region, the strongest candidates for the neurodevelopmental phenotypes that we identified are NDE1 and NTAN1, whose inactivation was reported as associated with abnormal neurological features [13-15].

The 16p13.11 microdeletion is associated with neurodevelopmental phenotypes such as autism, intellectual disability, epilepsy, and learning difficulties, and with non-CNS phenotypes such as physical dysmorphisms and congenital anomalies [1-5,7]. Recently, evidence of a male-biased autosomal effect has emerged; males with the microdeletion are more likely to manifest symptoms [16]. This microdeletion has also been found in normal individuals. The presence of this rearrangement in unaffected relatives may be due to a number of factors, such as incomplete penetrance, variable expressivity, or imprinting [17].

Our patient also carried a 625.8Kb interstitial triplication at 19p13.3 band. Triplications are rare chromosomal aberrations and are sometimes observed as the consequence of non-allelic homologous recombination [18]. Triplications of the short arm of chromosome 19 have never been reported.

The phenotype of our patient is comparable with that reported in other patients with a16p13.11 deletion, however our patient has more severe features such as brain abnormalities, brachycephaly, and bilateral talipes equinovarus. Also his neurological features seem to be more complicated than those previously reported in the literature.

Regarding 19p13.3 triplication, it is difficult to determine its effect on the phenotype of our patient. To our knowledge, there are no reported cases of triplications in literature and only few cases of duplications.

Ishikawa et al., [19] reported on four patients with constitutional interstitial 19p13.3 duplication, none of them overlapping our case. In the Decipher database, there are no patients with duplications encompassing the same region as that reported here, though there are three patients affected by intellectual disability with smaller, partially overlapping duplications (cases 258703, 271079, 253689).

Interestingly, our patient inherited the triplication from his mother, who presented mild intellectual disability and language delay. Among the genes included in the triplicated region, two genes, *SAFB1* and *SAFB2,* are probably of interest; they function as oestrogen receptor co-repressors, and their overexpression results in inhibition of proliferation also in the central nervous system [20].

Another interesting gene is *PTPRS*, which is expressed selectively and at high levels in the central and peripheral nervous systems during neural development. Its product plays a role in neuronal survival, synaptic plasticity, axon guidance, and nerve regeneration [21-23]. Furthermore, studies in chickens showed that overexpression of PTPRS in primary sensory neurons inhibits neurite outgrowth [24].

## Conclusions

In conclusion, we report on a boy with a combination of 16p13.11 microdeletion and a 19p13.3 triplication. The synergistic effect of the two genomic imbalances is possibly responsible for the complicated clinical features observed in our patient and hopefully our case will help us better understand the relative contributions to the phenotype of different chromosome imbalances and will enable us to provide better genetic counselling in the future. However, we cannot exclude that the patient’s phenotype could also be the result of a mutation in a specific gene.

## Methods

Standard GTG banding was performed at a resolution of 400–550 bands on metaphase chromosomes from peripheral blood lymphocytes of the patient and his parents. Molecular karyotyping was performed on the patient, his parents, maternal grandmother and maternal aunt using Human Genome CGH Microarray Kit G3 180 (Agilent Technologies, Palo Alto, USA) with ~13Kb overall median probe spacing. Labelling and hybridization were performed following the protocols provided by the manufacturers. A graphical overview was obtained using the Agilent Genomic Workbench Lite Edition Software 6.5.0.18. FISH was performed using Agilent Custom FISH 19p13.3 spanning 626Kb (chr19:5,015,926-5,641,995) (Green) (Agilent Technologies, Palo Alto, USA) and subtelomeric probe 19q (Orange) (Cytocell Aquarius, Cambridge, UK).

### Consent

Written informed consent was obtained from the patient for publication of this paper and any accompanying images. A copy of the written consent is available for review by the Editor-in-Chief of this journal.
